# Targeting NMDA receptors in stroke: new hope in neuroprotection

**DOI:** 10.1186/s13041-018-0357-8

**Published:** 2018-03-13

**Authors:** Qiu Jing Wu, Michael Tymianski

**Affiliations:** 1 Krembil Research Institute, University Health Network, 60 Leonard St, Toronto, ON M5T2S8 Canada; 2 Department of Physiology, University of Toronto, Toronto, ON Canada; 3 Division of Neurosurgery, University of Toronto, Toronto, ON Canada

**Keywords:** Ischemic stroke, NMDA receptors, Excitotoxicity, Death signaling complexes, Neuroprotection

## Abstract

NMDA (N-methyl-d-aspartate) receptors (NMDARs) play a central role in excitotoxic neuronal death caused by ischemic stroke, but NMDAR channel blockers have failed to be translated into clinical stroke treatments. However, recent research on NMDAR-associated signaling complexes has identified important death-signaling pathways linked to NMDARs. This led to the generation of inhibitors that inhibit these pathways downstream from the receptor without necessarily blocking NMDARs. This therapeutic approach may have fewer side effects and/or provide a wider therapeutic window for stroke as compared to the receptor antagonists. In this review, we highlight the key findings in the signaling cascades downstream of NMDARs and the novel promising therapeutics for ischemic stroke.

## Introduction: stroke epidemiology and need for effective therapeutics

Stroke is the second most common cause of death and the third most common cause of disability worldwide. In 2010, about 10% of all deaths and 4% of DALYs lost (disability adjusted life years) were caused by stroke [1]. It consumes near 4% of total health care costs each year and creates a huge burden on the health care system [2]. With an aging global population, the mortality rate and burden due to stroke will keep increasing. By 2030, stroke is estimated to cause 12 million deaths, and more than 200 million DALYs lost globally [1].

The two main types of stroke are ischemic and hemorrhagic. Ischemic strokes comprise about 87% of all strokes [2]. Ischemic stroke arises from a thrombotic or embolic blockage of brain arteries resulting in limited blood flow to the affected brain tissue, followed by energy depletion. This triggers a series of complex pathophysiological events including the disruption of ionic homeostasis, accumulation of synaptic and extrasynaptic glutamate, ion channel dysfuntion, membrane and DNA damage, inflammation and so on, eventually lead to neuronal cell death and ischemic brain injury [3–6].

So far the only FDA-approved pharmacotherapy for acute stroke is with intravenous thrombolytic therapy using recombinant tissue plasminogen activator (rtPA) [7, 8]. However, this agent has a 3–4.5 h therapeutic window, and risks producing an intracerebral hemorrhage (6–7% cases). This has limited the use of rtPA to only about 5% of all stroke patients [2, 9–11]. Thus there remains a significant unmet medical need for identifying more effective and safer stroke drugs.

For the past decades, extensive research has advanced our understanding of the stroke pathology. Excitotoxicity mediated by N-methyl-D-aspartate (NMDA) type of glutamate receptors has been at the center stage of stroke research. In this review, we highlight recent key findings in ischemic cell death signaling pathways linked to or downstream of NMDARs and newly developed drug candidates that act as neuroprotectants, agents that reduce the vulnerability of ischemic brain to ischemia.

## Understanding stroke: excitotoxicity and NMDA receptors

Excitotoxicity is among the first identified, and most intensively studied ischemic cell death mechanism. The term “excitotoxicity” describes the process in which excess quantities of the excitatory neurotransmitter glutamate over-activates NMDARs and induces neuronal toxicity [12–14]. This has been considered as one of the major pathogenic mechanisms underlying ischemic brain injury [4, 15, 16].

During ischemia, restricted cerebral blood flow depletes the supply of oxygen and nutrients that are required by neurons to maintain ionic homeostasis [4]. Disrupted ionic gradients depolarize the cell and, among other things, trigger the release of excitatory neurotransmitters, namely glutamate, into the synaptic space. At the same time, energy depletion also impairs the function of re-uptake transporters so they are unable to clear excess glutamate. This results in the accumulation of excitatory glutamate in the extracellular space and the consequent over-activation of glutamate receptors of post-synaptic neurons.

Ionotropic glutamate receptors are ligand-gated ion channels that allow rapid ion influx in response to glutamate and comprise the gateway to excitotoxicity [17–20]. They contain both an extracellular glutamate binding site and a transmembrane ion channel. The two main subtypes of ionotropic glutamate receptors are NMDA (N-methyl-d-aspartate) receptors (NMDARs) and AMPA (α-amino-3-hydroxy-5-methylisoxazole-4-propionic acid) receptors (AMPARs). At the resting state, the channel pores of NMDARs are normally blocked by Mg^2+^. When glutamate is released from pre-synaptic sites, activated AMPARs cause a partial depolarization in the post-synaptic membrane sufficient to remove the Mg^2+^ block from NMDARs. Once NMDARs are activated, they flux Na^+^ and Ca^2+^ into the cell. The Ca^2+^ influx through NMDARs is not only critical for the normal physiological processes in neurons, but also plays a major role in initiating ischemic cell death [17–19, 21]. In excitotoxicity, excess glutamate release results in over-activation of NMDARs and leads to calcium overload inside the neurons. Calcium overload triggers a range of downstream pro-death signaling events such as calpain activation [22, 23], reactive oxygen species (ROS) generation [24–26], and mitochondrial damage [4, 24, 27], resulting in cell necrosis or apoptosis.

Given the pivotal role of NMDAR in excitotoxicity, the initial therapeutic approach was to block the receptors [4, 7, 28]. NMDAR antagonists were designed to target different sites: non-competitive antagonists that block the ion channels, competitive antagonists that prevent excitatory neurotransmitters from binding to the glutamate recognition site, and glutamate release inhibitors that blocked presynaptic voltage sensing Na^+^ channels [29]. In pre-clinical studies in rats, NMDAR antagonists protected neurons from ischemic death in a model of middle cerebral artery occlusion (MCAO). The MCA can be occluded either transiently or permanently in these models, producing strokes of various severity [30–33]. However, despite initial promise in rodents such as rats, NMDAR antagonists have failed to be translated for clinical use in acute stroke [6, 34]. The explanation for these failures of translation is likely multi-factorial [7]. Two important drawbacks are the short therapeutic time window, and dose-limiting safety concerns [16, 29, 35]. The NMDAR antagonists have to be administered either before or immediately after stroke to be effective [7, 35, 36]. In addition, the NMDAR antagonists can cause severe side effects such as nausea, vomiting, cardiovascular and psychomimetic effects in treated patients [35, 37–39]. In retrospect it appears that NMDAR blockade will interfere with normal neuronal function and cause substantial side effects at potentially therapeutic doses.

Due to the lack of clinical success with NMDA receptor antagonists, the focus of stroke neuroprotection shifted towards the identification of downstream intracellular signaling pathways triggered by NMDARs.

## NMDA receptors: dual roles in neuronal survival and death

**Fig. 1:**
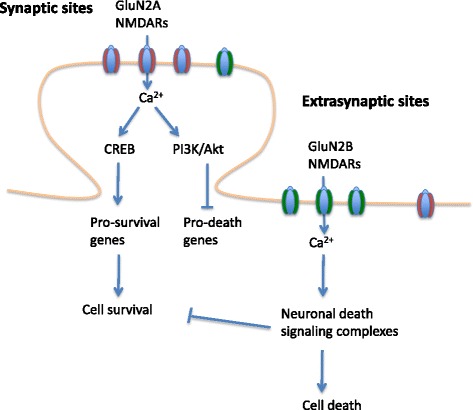
Dual roles of NMDARs in cell survival and death. Activation of NMDARs can trigger pro-survival or pro-death signaling depending on the subcellular locations or subtypes of NMDARs. In mature neurons, GluN2A-containing NMDARs are abundant in the synapses, and GluN2B-containing NMDARs are enriched in the extrasynaptic sites. In general, synaptic/GluN2A-containing NMDARs are associated with pro-survival effects, whereas extrasynaptic/GluN2B-containing NMDARs are linked to pro-death signaling complexes

Structurally, NMDARs are heterotetramers formed by two GluN1 subunits and two glutamate binding GluN2 subunits. The GluN2 subunits can be GluN2A-GluN2D, as well as GluN3A and GluN3B, all of which have distinguishing properties and expression patterns in the CNS [40]. The most widely expressed NMDARs contain GluN1 subunits in combination with either GluN2B or GluN2A. NMDARs play central roles in synaptic plasticity, brain development, learning and memory [41, 42]. However, when excessively activated in ischemic stroke, NMDARs initiate toxic cascades that kill the neurons. Recent studies suggest that the dual roles of NMDARs in neuronal survival and death may depend on the subcellular locations and subtypes of the receptors that are activated [16, 43–46] (Fig. 1).

In the receptor location hypothesis, stimulating synaptic NMDARs activates pro-survival signaling pathways, whereas the activation of extrasynaptic NMDARs is associated with pro-death pathways. Synaptic NMDAR stimulation activates the PI3K (Phosphoinositide-3-kinase)/AKt kinase pathway, CREB (cAMP-response element binding protein)-dependent gene expression and suppression of pro-death genes, all of which contribute to pro-survival effects [46]. Upon NMDAR opening, PI3K is activated by Ca^2+^ and calmodulin that phosphorylate membrane phospholipid PtdIns(4,5)*P*2) to PtdIns(3,4,5)*P*3 [47]. PtdIns(3,4,5)*P*3 interacting kinase PDK1 (phosphoinositide dependent protein kinase1) is then recruited to the membrane and activates Akt by phosphorylation [48]. Akt promotes cell survival by phosphorylating a number of downstream targets. It inactivates GSK3β (glycogen synthase kinase 3β), pro-apoptotic *Bcl-2* associated death promotor BAD [49], JNK (c-Jun N-terminal Kinase)/p38 activator ASK1 (apoptosis signal-regulating kinase 1) [50], and apoptotic p53 [51]. Synaptic NMDAR activation also induces the expression of pro-survival genes. Synaptic NMDAR activity and Ca^2+^ influx activates the Ras/ERK (extracellular signal regulated kinase) signaling and nuclear CAMKs (Ca^2+^/calmodulin dependent protein kinases), which then phosphorylates and activates CREB [52, 53]. Activation of CREB induces the expression of pro-survival genes that protect the neurons against apoptotic insults. CREB target genes include anti-apoptotic *BTG2*, apoptotic p53 suppressor *BCL6*, and survival promoting neurotrophin BDNF (brain derived neurotrophic factor) [44, 46].

In contrast with the pro-survival effect of synaptic NMDAR activities, extrasynaptic NMDARs are associated with pro-death signaling pathways. The activated extrasynaptic NMDARs attenuate the pro-survival signaling mediated by the synaptic NMDARs. For example, the activation of extrasynaptic NMDARs dephosphorylates and inactivates CREB [44]. They also dephosphorylate and inactivate ERK pathway, which prevents the activation of CREB and promote the expression of pro-death genes [46, 54]. Weak NMDAR antagonists such as memantine can selectively block extrasynaptic NMDARs, suggesting that there is a potential to modulate the balance between pro-survival and pro-death signaling in ischemic stroke [55, 56].

In addition, different NMDAR subunit combinations (receptor subtypes) may recruit different downstream signaling complexes resulting in distinct functional effects. GluN2A- and GluN2B-containing NMDARs are the two predominant types of NMDARs in the adult forebrain. During early development, GluN2B-containing NMDARs are abundant in the prenatal brain and then decreases postnatally, while the expression of GluN2A-containing NMDARs increases with development [40]. In the adult brain, GluN2B-containing NMDARs are enriched in the extrasynaptic sites, whereas GluN2A-containing NMDARs are highly expressed at the synapse. The GluN2A- and GluN2B- containing NMDARs also play different roles in response to ischemic insults: activation of either synaptic or extrasynaptic GluN2B-containing NMDARs results in excitotoxicity and neuronal apoptosis, whereas activation of synaptic or extrasynaptic GluN2A-containing NMDARs leads to neuronal survival and neuroprotection against ischemic insults [57, 58].

Given the dual roles of NMDARs, it would be ideal to selectively inhibit only the pro-death signaling from the receptors and not interfere with pro-survival pathways. One approach could be the targeting of extrasynaptic/GluN2B-containing NMDARs. However, the segregation of the different NMDAR subunits among synaptic vs. extrasynaptic sites is not absolute, hence blocking the extrasynaptic GluN2B-containing NMDARs may still antagonize synaptic GluN2A-containing NMDARs [5].

## Targeting NMDAR pro-death pathways: potential therapeutics

An alternative to selectively targeting GluN2B- containing NMDARs may be to selectively target pro-death mechanisms downstream of NMDARs. This approach has shown significant promise in neuroprotection.

### GluN2B-PSD95-nNOS complex

**Fig. 2:**
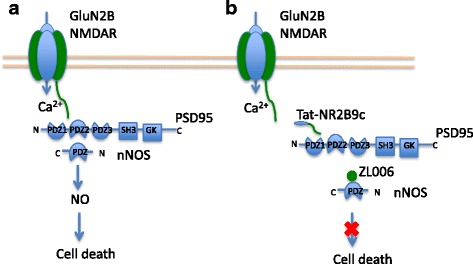
Perturbing the GluN2B-PSD95-nNOS complex protects neurons from ischemic injury. **a** The activity of GluN2B-containing NMDARs is linked to the downstream nNOS and production of NO through the scaffolding protein PSD95. Over-activation of NMDARs in excitotoxicity produces a toxic level of NO and leads to neuronal death. **b** Interfering peptides and small molecules disrupting the protein complex can reduce NO production and prevent stroke damage

A well-characterized death-signaling pathway in ischemic stroke is found in the multi-protein complex associated with membrane-bound NMDARs. It is the GluN2B-PSD95-nNOS pathway, in which the scaffolding protein postsynaptic density-95 (PSD95) links NMDARs to downstream molecules including nitric oxide synthase (nNOS). PSD95 contains three PDZ domains (an acronym derived from post synaptic density protein-95, drosophila disc large tumor suppressor-1, and zonula occludens-1 protein-protein interaction domains). The PDZ1 and PDZ2 domains of PSD95 bind directly to the threonine/serine-X-valine-COOH (T/SXV) motif at the intracellular C-termini of GluN2 NMDAR subunits [59]. The PDZ2 domain of PSD95 also binds to the N-terminus of nNOS [60]. This molecular organization allows Ca^2+^ influx from over-activated NMDARs to cause overactivation of nNOS, which then produces nitric oxide (NO), a reactive nitrogen species and a known effector of excitotoxicity [61]. Disrupting the GluN2B-PSD95-nNOS complex suppresses NMDAR-mediated NO production and protects neurons from excitotoxicity [61–64] (Fig. 2).

#### Downstream of the complex: NO mediates neuronal death

NO reacts with superoxide free radicals to form the highly reactive oxidant peroxynitrite. That can cause protein oxidation, lipid peroxidation, and DNA damage [65–67]. Peroxynitrite mediated DNA damage can also activates poly (ADP)-ribose polymerase (PARP-1), a nuclear DNA repair enzyme, causing energy deprivation of ATP and NAD and triggering the mitochondrial release of apoptosis inducing factor (AIF) [26, 68, 69]. AIF then translocates into the nucleus and causes DNA fragmentation and cell death.

#### Clinical success of the PSD95 inhibitor Tat-NR2B9c (NA-1)

One approach to disrupting the production of NO in excitotoxicity is by using interfering peptides that bind either PSD95 or nNOS, thereby perturbing the ability of NMDAR activity to activate nNOS. One such interfering peptide had been termed “Tat-NR2B9c or NA-1”, and is comprised of the 9 C-terminal residues of the GluN2B subunit fused with 11 residues of the cell membrane transduction facilitator Tat. Tat-NR2B9c was shown to uncouple NMDARs from PSD95 and attenuate downstream neurotoxic signaling [61, 70, 71] (Fig. 2). A number of in vivo studies in rats have demonstrated the neuroprotective effects of Tat-NR2B9c in reducing infarct volume and improving neurobehavioral outcomes when administered after ischemic stroke [61–63, 72].

To bridge the translational gap between rat animal models and human clinical trials, experiments were conducted to examine the effect of Tat-NR2B9c after MCAO in non-human primates with genetic, anatomic, and behavioral similarities to humans [64]. These experiments showed that stroke damage can be prevented in non-human primates in which a Tat-NR2B9c is administered after stroke onset in experimental paradigms that were designed to mimic clinically relevant situations. The treatment reduced infarct volumes as gauged by magnetic resonance imaging and histology, preserved the capacity of ischemic cells to maintain gene transcription in genome-wide screens of ischemic brain tissue, and significantly preserved neurological function in neuro- behavioral assays. These results show that the strategy of targeting PSD95 rather than NMDARs can reduce stroke damage in human-like brains, suggesting promise for future clinical use.

A clinical proof-of-concept study of NA-1 has been completed to assess whether NA-1 could reduce ischemic brain damage in human beings. This was a double-blind, randomized, controlled study conducted at 14 hospitals in Canada and the USA. The study enrolled patients who had a ruptured or unruptured intracranial aneurysm amenable to endovascular repair, as up to 90% of human beings undergoing endovascular intracranial aneurysm repair show small, embolic, procedurally induced ischemic strokes on diffusion-weighted (DWI) MRI. One hundred eighty-five patients were randomized to receive either NA-1 or saline control at the end of their endovascular procedure [71, 73]. Patient demographics, medical risks, adverse events and procedures were balanced between the groups. Patients who received NA-1 sustained fewer ischemic infarcts as gauged by MRI imaging. Among patients with ruptured, NA-1 treatment reduced the number and volume of strokes by all MRI criteria and improved neurological outcome. Thus, the strategy of treating a stroke with an agent that targets PSD95 after ischemia has begun has clinical promise.

#### Small molecules targeting the complex: ZL006, IC87201

Recent studies have discovered two small molecules ZL006 and IC87201 that are also reported to dissociate the GluN2B-PSD95-nNOS complex. A de novo small molecule ZL006 was synthesized to selectively inhibit the ischemia induced PSD95 and nNOS interaction (Fig. 2). This molecule showed neuroprotective effects in vitro and reduced cerebral ischemic injury in mouse and rat stroke models [74]. In addition, ZL006 is reported to cross the blood brain barrier and to not affect the normal function of NMDARs and nNOS. A similar compound IC87201 was discovered by *Florio* et al. using high throughput screening [75]. It was reported to disrupt the pathogenic PSD95-nNOS interaction without inhibiting the normal nNOS activity in neurons [75]. IC87201 has been tested for its anti-nociceptive effects, and was reported to reduce NMDA-induced hyperalgesia in mice, though its neuroprotective potential in stroke remains to be tested. Recent studies have challenged whether either of these molecules actually interact with the PDZ domains of nNOS or PSD-95, or inhibit the nNOS-PDZ/PSD-95-PDZ interface [76].

#### Peroxynitrite scavengers and antioxidants

The neuroprotective efficacy of peroxynitrite scavengers such as disufenton sodium (NXY-059) has been evaluated in rodent stroke models as well as in marmosets [77, 78]. However in a pivotal clinical trial, NXY-059 failed to show efficacy [79].

Uric acid is a powerful scavenger of free radicals in plasma [80]. Uric acid has been shown to attenuate peroxynitrite-mediated damage and alleviate ischemic injury in rodent stroke models [8, 81–83]. It also showed synergistic neuroprotection with thrombolytic agent rtPA (alteplase) in preclinical studies [82, 84]. The safety and efficacy of uric acid with thrombolytic therapy have been assessed in the phase 2b/3 URICOICTUS trial [85]. Although the combination of uric acid and rtPA did not prove efficacy in the primary outcome (modified Rankin score at 90 days follow-up), the treatment did not lead to safety concerns [8, 85]. In addition, the uric acid treatment was found to improve functional outcome in patient subgroups [8, 85–87]. More clinical trials studying the efficacy of uric acid are currently on going. In a recent study, the combined treatment of uric acid and rtPA prevented early ischemic stroke progression after acute ischemic stroke [84].

Edaravone is another anti-oxidant drug that scavenges hydroxyl, peroxyl, and superoxide radicals. It has been marketed in Japan since 2001 to treat acute ischemic patients within 24 h of stroke attack [88]. Edaravone was shown to reduce blood brain barrier dysfunction, reduce brain edema, decrease cortical infarct size, and decrease behavioral deficits in rodent and rabbit stroke models [88–92]. A recent review assessed clinical studies during years 1993–2008 has suggested that Edaravone may be a useful therapeutic treatment for ischemic stroke, but the efficacy of Edaravone should be further tested in randomized controlled clinical trials with standardized dosage, treatment time and duration [88].

### GluN2B-DAPK1 interaction

DAPK1 (death-associated protein kinase 1) is a Ca^2+^/calmodulin (CaM) dependent serine/threonine protein kinase whose activity is associated with apoptotic cell death [93]. DAPK1 is highly expressed in the brain. At basal condition, DAPK1 activity is suppressed by autophosphorylation at serine 308 in the CaM regulatory domain. Upon binding with Ca^2+^ activated CaM, the catalytic activity of DAPK1 is disinhibited and the pro-apoptotic activity is stimulated [94, 95]. In ischemic stroke, the over-activation of NMDAR leads to excessive Ca^2+^ influx into the cell and activates CaM and the calcinerin phosphatase (CaN), which in turn dephosphorylate and activate DAPK1 [96].

**Fig. 3:**
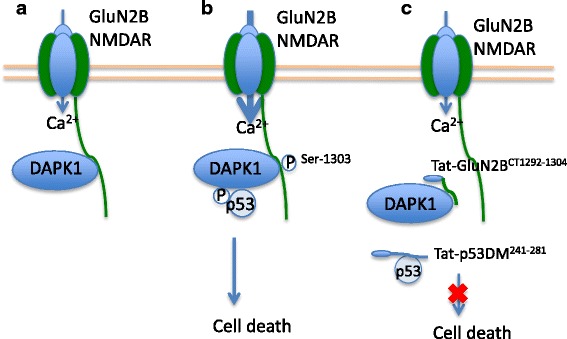
Disrupting GluN2B-DAPK1-p53 complex prevents ischemic damage. **a** Under ischemic condition, excitotoxic stimulation of GluN2B-containing NMDARs activate and recruit DAPK1 to the C-terminus of GluN2B. **b** Activated DAPK1 phosphorylate GluN2B to enhance the currents through GluN2B-containing NMDARs. On the other hand, activated DAPK1 also directly binds and phosphorylates p53 to mediate neuronal death. **c** Disrupting the complex by the interfering peptides protected neurons from ischemic cell death

A recent study by Tu et al. demonstrated that activated DAPK1 is recruited to the GluN2B subunit of NMDARs after ischemic insults [97]. DAPK1 directly binds to amino acids 1292–1304 at the intracellular carboxyl tail region (GluN2B^CT^) of the GluN2B subunit. DAPK1 activation increases phosphorylation at site Ser-1303 within the DAPK1 binding domain of GluN2B subunit, and enhances GluN2B-containing NMDAR channel conductance [97] (Fig. 3). Based on Tu et al.’s findings, GluN2B-DAPK1 may play an important role in mediating ischemic damage. However, a more recent research by McQueen et al. has challenged previous report by Tu et al. [98] McQueen et al. observed that DAPK1 gene deletion did not protect neurons from excitotoxic and ischemic insults. The discrepancies between the two studies may need future investigation.

#### Development of Tat-GluN2B^CT1292–1304^

Tu et al. has developed an interfering peptide Tat-GluN2B^CT1292–1304^ to uncouple DAPK1 from the GluN2B subunit (Fig. 3). The administration of GluN2B^CT1292–1304^ attenuates Ca^2+^ influx through extrasynaptic NMDARs and protects neurons from ischemic cell death in vivo, suggesting the therapeutic potential against ischemic injury. On the other hand, the recent study by McQueen et al. suggested that both Tat-GluN2B^CT^ and scrambled peptide Tat-GluN2B^CT^ are direct NMDAR antagonists [98]. The mechanism of action and the therapeutic potential of tat-GluN2B^CT^ may require future clarification.

#### Downstream of DAPK1: Tat-p53DM^241–281^

One of the substrate for the DAPK1 kinase is the tumor suppressor p53, a transcriptional regulator that controls the cell death pathways in ischemic stroke and neurodegenerative diseases. Recently, Pei et al. found that activated DAPK1 phosphorylates p53 via direct protein-protein interaction [99]. The death domain of DAPK1 (DAPK1DD) directly binds to the p53 DNA binding motif consists of amino acids 241–281. The authors showed the significance of DAPK1-p53 interaction in mediating necrotic and apoptotic cell death [95, 99]. Based on this knowledge, an interfering peptide Tat-p53DM^241–281^ was constructed to disrupt the interaction between DAPK1 and p53 (Fig. 3). Tat-p53DM^241–281^ specifically inhibits the downstream signaling cascade of DAPK1, including p53-mediated expression of pro-apoptotic genes *Bax* and *Puma*, and apoptotic mediator caspase-3 [99]. In addition, Tat-p53DM^241–281^ reduced infarct volume, and improved neurobehavioral outcomes even when administered 6 h after MCAO [100]. The long therapeutic time window of Tat-p53DM^241–281^ makes it a potentially promising candidate for stroke treatment.

### GluN2B NMDAR-PTEN

Phosphatase and tensin homolog deleted on chromosome ten (PTEN) is an important tumor suppressor with lipid and protein phosphatase activity. Previous research identified the involvement of PTEN in neuronal death after ischemia [101, 102]. PTEN can mediate apoptotic cell death by dephosphorylating phosphatidylinositol 3,4,5-trisphosphate (PIP3) and inhibiting the pro-survival Phosphatidylinositol-3-kinase (PI3K)/Akt signaling cascade [103, 104].

Once activated by the calcium influx through NMDARs, PTEN can be recruited to the neuronal death complex associated with the GluN2B-containing NMDARs. It directly interacts with the GluN1 subunit of GluN2B-containing NMDARs. This interaction augments the channel currents flow through GluN2B-containing NMDAR channel pores and further enhances the recruitment of PTEN to the GluN2B subunit mediated death-signaling complex. It is recently identified that excitotoxic stimulation of NMDARs can induce the PTEN nuclear translocation, which results in a marked reduction in pro-survival nuclear PIP3 and Akt phosphorylation [102, 105]. Increased nuclear PTEN accumulation and PTEN’s cell death promoting activities contribute to the NMDAR mediated neuronal death in excitotoxicity.

#### Blocking PTEN nuclear translocation by Tat-K13

PTEN nuclear translocation is enabled by a single ubiquitination at residue K13 in neurons under excitotoxic stress [105]. In order to disrupt this cell death signaling, an interfering peptide Tat-K13 was developed. It consists of the transmembrane domain Tat protein and amino acids flanking the K13 ubiquitination site of PTEN [105]. Rats treated with Tat-K13 in an ischemic model had significantly reduced stroke lesion size even when administered 6 h after the stroke onset compared to the Tat-K289 control group [105]. The neuroprotective effect of Tat-K13 at 6 h supports the concept that disrupting the downstream pro-death signaling cascade can provide a wider therapeutic time window than blocking the upstream NMDAR channels.

### NMDAR-SFK-Panx1

The pannexin (Panx) family of ion channels belongs to the gap junction superfamily. The intracellular gap junction channels form connexins that are permeable to a wide range of ions, second messengers and metabolites. Thompson et al. first discovered that pannexin channels were involved in anoxic depolarization and subsequent neuronal death under an ischemic condition OGD (oxygen glucose deprivation) [106–108]. Recently the same group showed NMDARs, Src kinases (SFK) and Pannexin-1 (Panx1) form a signaling complex in mediating ischemic injury [109, 110]. During ischemia, NMDAR activates SFKs, which in turn phosphorylates site Y308 in the C-terminal of Panx1 to activate Panx1 and induce secondary ischemic currents [108, 110].

#### Block Panx1 phosphorylation by Tat-Panx_308_

Interfering peptide Tat-Panx_308_ resembles the C-terminal epitope of Panx1 including the Y308 site. Tat-Panx_308_ blocks the phosphorylation and activation of Panx1 by Src kinases during ischemia, and disrupts the NMDAR-Src-Panx1 complex [110]. Administration of Tat-Panx_308_ before or 2 h after stroke onset reduced lesion size and sensorimotor deficits in rats, demonstrating the neuroprotective effect of dissociating the complex [110].

### Further downstream death signaling proteins

#### Calpains: cleavage of NCX3, kidins220, STEP, mGluR1

**Fig. 4:**
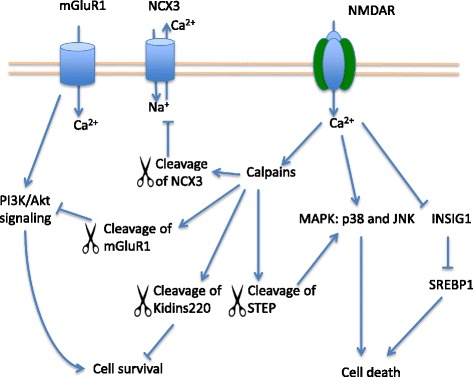
Further downstream cell death signaling proteins activated by NMDARs. Stimulation of NMDARs in excitotoxicity activates calpain-mediated cleavage of proteins and contributes to cell death. Examples of the substrates for calpain-cleavage include NCX3, mGluR1, Kidins220, and STEP. In excitotoxicity, NMDARs also activate p38 and JNK to induce cell death. In addition, NMDAR stimulation triggers the degradation of INSIG1 and disinhibits SREBP1-mediated cell death

Calpains are a family of calcium dependent cysteine proteases involved in NMDAR mediated excitotoxicity. Recent research suggests that stimulating the extrasynaptic subpopulation of NMDARs can activate calpains and induce cell death [22, 23, 111, 112] (Fig. 4). When activated, calpains can modulate substrate functions and regulate cellular mechanisms through substrate proteolysis. It’s remarkable that a novel calpain inhibitor SNJ-1945 demonstrated neuroprotection in cerebral ischemia in mice even when treatment was given 6 h post stroke [113].

#### Calpain cleavage of NCX-3

Excitotoxic calpain cleavage of plasma membrane sodium-calcium exchanger (NCX3) can induce calcium overload in the cytoplasm and mediate excitotoxic neuronal death. NCX is an important regulator of intracellular calcium level by removing Ca^2+^ from the cytoplasm. Following activation of NMDARs in excitotoxicity, NCX partially recovers the intracellular calcium concentration back to the physiological level [6, 114]. Inhibiting calpains or replacing NCX3 with another non-cleavable isoform NCX2 prevents calcium overload and neuronal death [115].

#### Calpain cleavage of Kidins220 and Tat-K

Kinase D-interacting substrate of 220 kDa (Kidins220) is involved in regulating and integrating signaling pathways that are essential for neuronal survival and function [116–118]. Kidins220 is involved in neurotrophin and ephrin receptors signaling [117, 118]. Excitotoxic stimulation of GluN2B-containing NMDARs activates calpains to truncate Kidins220, and impairs the neurotrophic signaling, evenally lead to ischemic neuronal damage [119].

To interfere with this process, a 25-amino acids peptide (Tat-K) was developed. It contains a short Kidins220 sequence enclosing the calpain cleavage site (AA1668–1681) linked to the Tat transmembrane protein [120]. Application of Tat-K in NMDA-treated neurons decreased calpain cleavage of Kidins220, preserved the activity of ERK and CREB that are critical for neuronal survival, and promoted cell viability [120].

#### Calpain cleavage of STEP and Tat-STEP

One of the substrates for calpain cleavage is the striatal enriched protein tyrosine phosphatase (STEP) [23]. STEP is an intracellular tyrosine phosphatase that antagonizes the activity dependent strengthening of synapses [121]. It dephosphorylates and inactivates a number of important synaptic signaling proteins including two of the mitogen activated protein kinases (MAPK): the extracellular signal-regulated kinase (ERK), and stress response protein kinase p38 [122, 123]. STEP was also shown to dephosphorylate GluN2B subunit at Tyr1472 and facilitates the internalization of GluN2B-containing NMDARs [124]. Activated synaptic NMDARs degrade STEP and promote pro-survival ERK signaling. In contrast, stimulating extrasynaptic NMDARs invokes calpain-mediated cleavage of STEP61 (full length protein) into STEP33 (cleavage product) [22, 23]. Truncated STEP loses its ability to bind and dephosphorylate the protein targets including p38 and GluN2B subunit of NMDARs that are enriched in the extrasynaptic region. The loss of function of STEP after calpain cleavage enhances p38 activity and prevents the endocytosis of GluN2B containing NMDARs, which contribute to ischemic damage and neuronal death.

As the activation of extrasynaptic NMDARs induces calpain mediated cleavage of STEP and causes cell death, an interfering peptide consisting of 16 amino acids spanning the cleavage site of STEP fused with TAT was developed [23]. Tat-STEP is reported to prevent the NMDAR mediated cleavage of STEP by calpains, reduces consequent p38 activation, and protects neurons from ischemic cell death in vitro [23, 125].

#### Calpain cleavage of mGluR1 and Tat-mGluR1

The activation of NMDARs in excitotoxicity and subsequent activated calpains have also been linked to the cleavage of metabotropic glutamate receptor 1 (mGluR1). Native mGluR1 interacts with the adaptor protein Homer and nuclear Phosphoinositide 3 kinase enhancer (PIKE) complex to activate the pro-survival PI3K/Akt signaling pathway and to protect neurons from apoptosis [126]. The calpain-mediated cleavage of mGluR1 converts the receptor from pro-survival into pro-death signaling in ischemia [6, 23]. Activation of NMDARs triggers calpains to truncate mGluR1 at Ser936 in the C-terminal domain [127]. The truncated mGluR1 is unable to activate the neuroprotective PI3K/Akt signaling pathway while its ability to increase cytosolic calcium remains intact [127].

To selectively block calpain-mediated cleavage of mGluR1, an interfering peptide was synthesized with an amino acid sequence spanning the calpain cleavage site and Tat protein transduction domain that renders the peptide permeable across cell membranes [127]. The interfering peptides compete with the endogenous mGluR1 for calpain truncation and protect the native mGluR1 receptors in neurons. Treatment with Tat-mGluR1 selectively reduced mGluR1 truncation at low concentrations (1-2uM), and prevented excitotoxic neuronal death in vitro and in vivo [127].

### MAPKs: p38 inhibitors, D-JNKI-1

The mitogen-activated protein kinase (MAPK) consists of a family of serine/threonine kinases that mediate intracellular signaling associated with cellular functions such as proliferation, survival and death [128–131]. The three most extensively studied subfamilies of MAPKs are: extracellular signal-regulated kinase 1/2 (ERK1/2); p38 MAPK; and c-Jun amino terminal kinase (JNK). ERK1/2 signaling is involved in CREB activation and mainly pro-survival [128]. In contrast, p38 and JNK are stress response proteins that activate death-related transcription and mediate neuronal apoptosis [128–130, 132].

P38 and JNK MAPKs have been implicated in the NMDAR-dependent neuronal apoptosis after stroke [133–135] (Fig. 4). P38 is activated by Rho, a member of the Rho family GTPases, and induces neuronal death following excitotoxic NMDAR activation [135]. As mentioned above, calpain cleavage of STEP is also involved in p38 activation and excitotoxic cell death [23]. In addition, p38 activation may be downstream of the GluN2B-PSD95-nNOS complex, and partially contributes to the death-promoting activity of the complex in excitotoxicity [6, 136, 137]. p38 inhibitor SB239063 prevented excitotoxic neuronal death in vitro and in vivo rat focal ischemic stroke model [133, 138–140].

JNK, also known as stress-activated protein kinase (SAPK), is activated in excitotoxicity and mediates neuronal death. Mice lacking JNK3, an isoform of JNK highly expressed in the brain, are resistant to excitotoxic neuronal apoptosis [141]. A peptide inhibitor Tat-JBD_20_ (also known as JNK inhibitor-1) was designed to block JNK from binding with its downstream substrates including c-Jun, which is a major target of JNK involved in stress-induced apoptosis [142]. JNK inhibitor peptide Tat-JBD_20_ has a Tat transporter sequence plus 20 amino acid JNK binding motif of JNK interacting protein-1/islet-brain 1 (JIP-1/IB1) [143–145]. The interfering peptide is synthesized in D-retroinverso form (D-JNKI-1) to prevent protease-mediated degradation in neurons and expand its half-life in vivo [145, 146]. The JNK inhibitor D-JNKI-1 has been shown to protected neurons in vitro and reduce neuronal damage in animals subjected to focal ischemic stroke [145]. D-JNKI-1 shows neuroprotection even when administered as late as 6 or 12 h after the stroke onset [145]. Late administration in transient ischemic animal model also reduced behavioral impairment up to 14 days [145].

### SREBP1: Indip

SREBP1 is a transcription factor and regulator for cholesterol, fatty acid, triglyceride, and phospholipid biosynthesis [147]. Recently SREBP1 has been identified as an NMDAR-dependent mediator of excitotoxic neuronal death following ischemic stroke [6, 16, 148] (Fig. 4). Under ischemic conditions, the activation of NMDARs induces ubiquitination and proteasome-mediated degradation of insulin-induced gene 1 (INSIG1) at the endoplasmic reticulum (ER). Native INSIG1 inhibits and retains SREBP1 in the ER. The degradation of INSIG1 enables SREBP1 to travel to the Golgi apparatus where SREBP1 is cleaved and becomes activated. The active SREBP1 then translocates into the nucleus and modifies gene transcriptions to mediate neuronal death.

To block this pathway, an interfering peptide Indip (INSIG1 degradation inhibiting peptide) has been developed to inhibit INSIG1 degradation. Indip contains a Tat-linked peptide with amino acid sequence flanking the two lysine-156 and 158 ubiquitination sites of INSIG1 that are required for cleavage [149]. It inhibited INSIG1 degradation, prevented SREBP1 activation and protected neurons from neuronal death in vitro and in vivo stroke models. Indip was neuroprotective when administered 2 h after stroke, and improved neurobehavioral outcomes for up to 7 days [148].

## Concluding remarks and future directions

NMDARs are essential in supporting neuronal functions under physiological functions, and also play a central role in excitotoxicity that causes neuronal death after ischemic stroke. Early treatments blocking NMDARs with antagonists failed to be translated into successful clinical neuroprotective therapies, mainly due to poor tolerance of the drugs and a short therapeutic time window. Because of the dual roles of NMDARs in pro-survival and pro-death signaling in neurons, NMDAR antagonism may eliminate survival signaling and impair neuronal function, resulting in severe adverse effects. Thus it would be better to selectively block only the pro-death effects of NMDARs while leaving pro-survival pathways intact. Moreover, once activated NMDARs trigger downstream pro-death signaling pathways, blocking the receptors may no longer be effective.

**Fig. 5:**
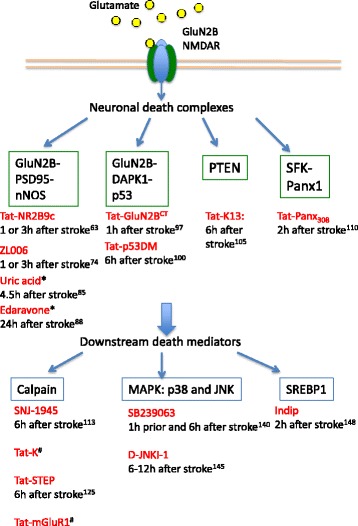
Summary of excitotoxic pathways, mediators and potential therapeutics. The highlighted neuronal death signaling pathways associated with excitotoxicity are: GluN2B-PSD95-nNOS, GluN2B-DAPK1-p53, GluN2B NMDAR-PTEN, and NMDAR-SFK-Panx1. Further downstream death mediators of excitotoxicity are calpain, MAPK:p38 and JNK, and SREBP1. The interfering peptides and molecules targeting each neurotoxic pathway/mediator are listed in red, and their time windows of administration after stroke onset were previously tested in animal stroke models or clinical trials. *:Peroxynitrite scavengers and antioxidants that may act downstream of the GluN2B-PSD95-nNOS pathway to prevent neurotoxicity. #: Therapeutic time window of the peptides not yet examined in animal ischemic stroke model. Numbers in superscript indicates references in the manuscript

Now our understanding of ischemic mechanisms is evolving. Recent research has identified several key signaling complexes and downstream effectors in mediating neuronal death in excitotoxicity. Based on this knowledge, interfering peptides and pharmacological inhibitors have been developed to specifically uncouple the neuronal death signaling from NMDARs without affecting the functional and survival signaling of the receptors (Fig. 5). In addition, because these new potential therapeutics target the downstream pathways of NMDARs, they may provide a wider therapeutic time window.

Given the new advancements in stroke research as discussed above, the relative importance and interplay among these signaling pathways still remains to be determined. In addition, combining multiple therapies that target different pathways in stroke may have a synergistic effect in neuroprotection. Future experiments may be conducted to test the safety and efficacy of combined treatments in preventing ischemic injury.

Furthermore, ischemic stroke and neurodegenerative diseases are commonly concurrent in patients [150, 151], suggesting an overlap of pathologies in neurological diseases. Therefore, a knowledge of ischemic cell death signaling and the identified neuroprotective candidates may also benefit the development of therapies for other neurological disorders.

## Abbreviations

AIF: Apoptosis inducing factor
AMPAR: α-amino-3-hydroxy-5-methylisoxazole-4-propionic acid receptors
ASK1: Apoptosis signal-regulating kinase 1
CAMKs: Ca^2+^/calmodulin dependent protein kinases
CREB: cAMP-response element binding protein
DAPK1: Death-associated protein kinase 1
ERK: Extracellular signal-regulated kinase
INSIG1: Insulin-induced gene 1
JNK: c-Jun N-terminal Kinase
Kidins220: Kinase D-interacting substrate of 220 kDa
MAPK: Mitogen activated protein kinases
MCAO: Occlusion of the middle cerebral artery
NCX3: Sodium-calcium exchanger
NMDAR: N-methyl-d-aspartate receptors
nNOS: Nitric oxide synthase
OGD: Oxygen glucose deprivation
PI3K: Phosphoinositide-3-kinase
PSD95: Postsynaptic density protein95
PTEN: Phosphatase and tensin homolog deleted on chromosome ten
rtPA: Recombinant tissue plasminogen activator
STEP: Striatal enriched protein tyrosine phosphatase
